# Using real-time location devices (RTLD) to quantify off-unit adult intensive care registrar workload: a 1-year tertiary NHS hospital prospective observational study

**DOI:** 10.1007/s10877-019-00383-z

**Published:** 2019-09-05

**Authors:** James Malycha, Daniel Murphy, Graham Barker, Guy Ludbrook, J. Duncan Young, Peter J. Watkinson

**Affiliations:** 1 Kadoorie Centre for Critical Care Research and Education, Nuffield Department of Clinical Neurosciences, University of Oxford, Level 3, John Radcliffe Hospital, Headley Way, Oxford, OX3 9DU UK; 2 Medical School, University of Oxford, Oxford, OX1 2JD UK; 3 Adult Intensive Care Unit, Oxford University Hospitals NHS Foundation Trust, Level 1, John Radcliffe Hospital, Headley Way, Oxford, OX3 9DU UK; 4 Faculty of Health and Medical Science, University of Adelaide, North Terrace, AHMS Floor 8, Adelaide, 5000 Australia

**Keywords:** Intensive Care Unit, Critical care, Medical workload, Real Time Location Device

## Abstract

UK national guidelines state deteriorating or at risk hospital ward patients should receive care from trained critical care outreach personnel. In most tertiary hospitals this involves a team led by an Intensive Care Unit (ICU) registrar. The ICU registrar must also review patients referred for possible ICU admission. These two responsibilities require work away from the ICU. To our knowledge the burden of this work has not been described, despite its importance in ICU workforce management and patient safety. A 12-month, prospective, observational study was carried out. The primary outcome measure was ICU registrar time spent on and off-unit. The study participants were senior and junior registrars on the rota of the 16 bed, Adult Intensive Care Unit at the John Radcliffe Hospital in Oxford. To measure their work patterns, this study used AeroScout ‘T2’ Real Time Location Device (RTLD) tags (Stanley Healthcare, Swindon). In our hospital, senior and junior ICU registrars spend roughly one-fifth of their time off-unit, half of which is spent in ED. This workload combines to leave the unit unattended at night up to 10% of the time. RTLDs provide a reliable, automated method for quantifying ICU registrar off-unit work patterns. This method may be adopted for quantifying other clinical staff work patterns in suitably equipped hospital environments.

## Introduction

Current guidelines stipulate that National Health Service (NHS) hospitals must have appropriate Intensive Care Unit (ICU) staffing to ensure safe on-unit and off-unit patient care [1]. The majority of ICU registrar work is generated on-unit through admitting, managing and discharging critically ill patients (a registrar is a junior doctor who has completed foundational training, usually 2 years, and is in training in a specialty area of medicine or surgery). However, the United Kingdom (UK) National Outreach Forum Operational Standards and Competencies for Critical Care Outreach Services state deteriorating hospital ward patients should receive care from trained critical care outreach personnel. In most NHS hospitals this involves a team led by an ICU registrar [2]. The ICU registrar must also review ward patients referred for possible ICU admission [3]. These two responsibilities require work away from the ICU. To our knowledge this work has not been quantified in the literature, despite its importance in ICU workforce management and patient safety. This study used an automated method to evaluate the work locations of ICU registrars within a tertiary NHS hospital over a 12-month period.

## Methods

The 12-month, prospective, observational study was carried out in accordance with the Revised Standards for Quality Improvement Reporting Excellence (SQUIRE 2.0) guidelines [4]. The study ran from April 1st 2017 to March 31st 2018.

### Intervention

Real-time Location Devices (RTLDs) are small portable devices that can communicate with wireless (wifi) data networks. This study used AeroScout ‘T2’ RTLDs, (Stanley Healthcare, Swindon). The RTLDs determine their position by triangulating the signals from wireless access points (WAPs) and pass this information to the AeroScout software. The location of each WAP is premapped into the AeroScout software, so the RTLD position within the hospital building can be determined. The RTLD location is updated every 5 min. RTLDs are usually used to determine the location of mobile assets (such as portable physiological monitors). In this study the RTLDs were attached to the “baton” pagers carried by the registrars which are handed on at the end of each shift.

### Measures

The primary outcome measure was ICU registrar time spent on and off the ICU. The study participants were registrars on the senior and junior tiers of the rota for the 16 bed, Adult Intensive Care Unit of the John Radcliffe Hospital, which is part of the Oxford University Hospitals National Health Service Foundation Trust (OUHNHSFT).

### Analysis

Data analysis was carried out using Python version 2.7 (Python Software Foundation, Wilmington, USA).

### Ethical considerations

Informed consent was not required, but all registrars were made aware of the study. Data were collected automatically in real time and securely stored within the hospital’s Aeroscout system. Access to the data was granted to JM only.

### Validation

The tags were tested during the study by placing them in predesignated locations and times and cross-referencing these with the location data.

## Results

**Fig. 1:**
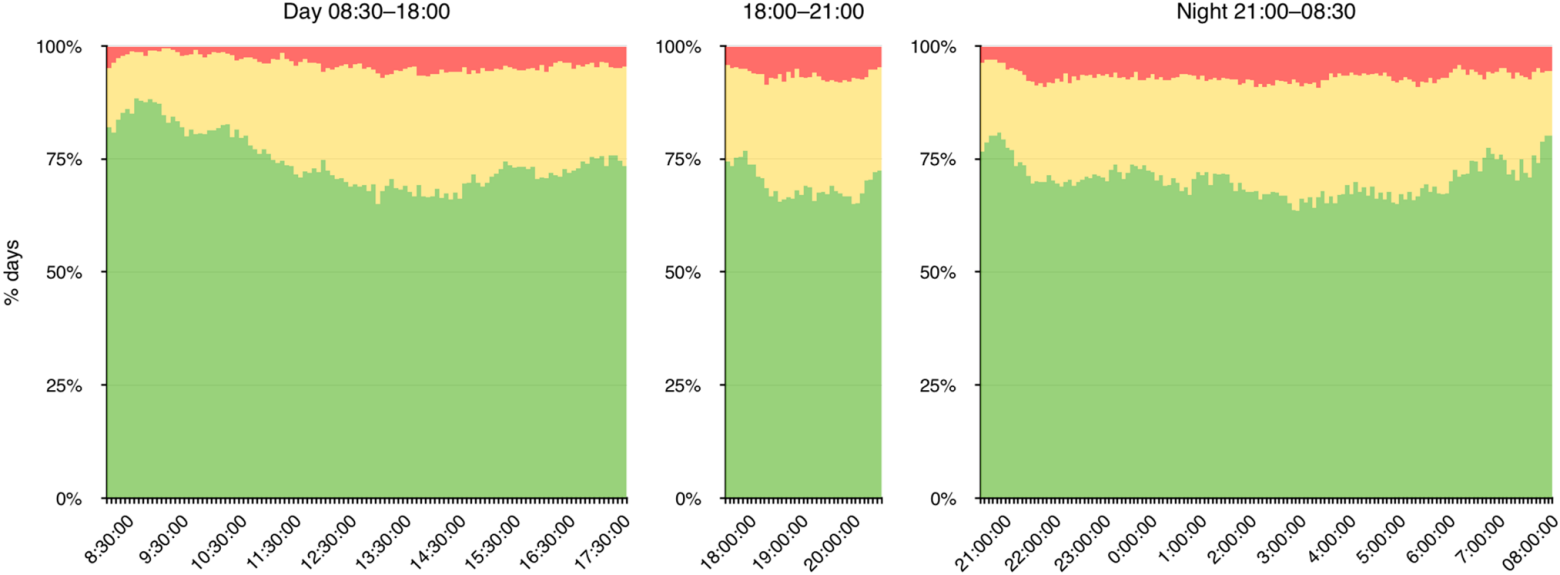
On-unit/off-unit analysis of ICU doctor location. x-axis: 24 h divided into 5 min increments y-axis:  % of days (mean) that the 5 min period had either none (red), one (amber) or both (green) doctors in the ICU (mean standard deviation is shown in the Appendix (Fig. 2))

Figure 1 shows the results of binary (i.e. on-unit/off-unit) analysis carried out on ICU doctor location during each of the shifts per 24 h. During the day shift (0830–1800) the adult ICU was staffed by both the senior and junior registrars as well as between two and five additional doctors of varying seniority. During the evening shift (1800–2100) and the night shift (2100–0830) the ICU was staffed by only the senior and junior registrars. In the latter two shifts, the ICU was therefore left unattended if both registrars were called off-unit.

**Fig. 3:**
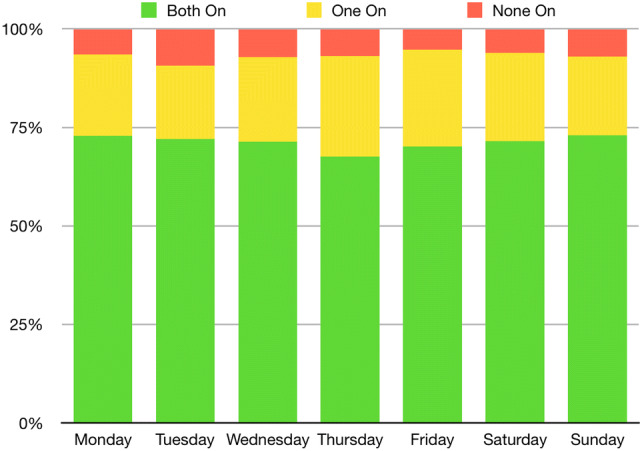
Tracker location variation between days of the week

**Fig. 4:**
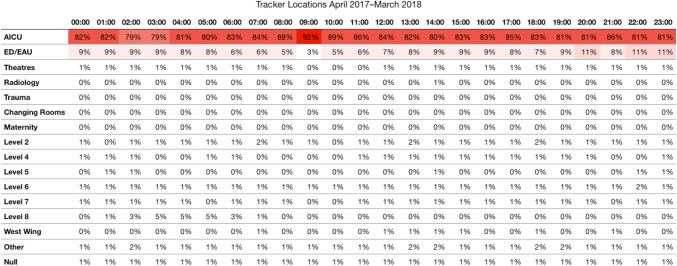

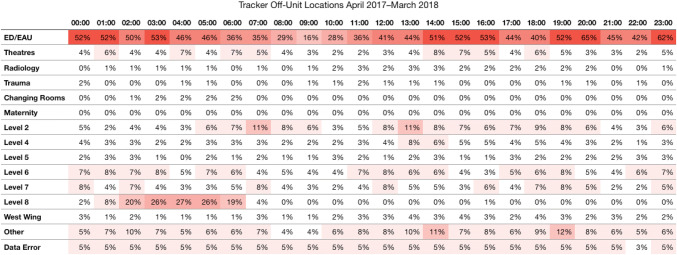
**a** Tracker locations (total). **b** Tracker locations (as a  % of time off-unit). Level 2: a mix of corridors, shops and outpatient departments, Levels 4 and 5: specialty wards, Level 6: surgical wards, Level 7: medical wards, Level 8: the on-call room for the Tier 2 registrars. West Wing: neurosurgical, plastics and ear, nose and throat surgical wards, null/data error: ‘floor hopped’ data

Off-unit location analysis showed that during the day shift (0800–1830), on average, the senior and junior registrars spent 7% of their time in the Emergency Department (ED)/Emergency Assessment Unit (EAU) and 8% in the general wards. During the evening shift (1830–2100), on average, both registrars spent 10% in the ED/EAU and 7% in the general wards. During the night shift (2100–0830), on average, both registrars spent 8% in the ED/AEU and 11% in the general wards. Variation in registrar off-unit work load between days of the week was minimal. Results are included in the Appendix (Fig. 3). A detailed description of the locations making up the category ‘general wards’ are included in the Appendix (Fig. 4a, b).

## Discussion

### Summary

The provision of intensive care in NHS hospitals increasingly involves off-unit registrar work, which is important but time consuming and expensive. We are the first to use RTLDs to quantify this activity. In our hospital, senior and junior ICU registrars spend roughly one-fifth of their time off-unit, half of which is spent in the ED. This workload combines to leave the unit unattended at night up to 10% of the time. This method and these data will help inform decisions about ICU off-unit workload and staffing and may in turn improve patient safety.

### Interpretation

This study established the feasibility of this method to establish staff working locations (both ICU and non-ICU) which could be applied to other hospitals. RTLD tags are already widely used in the NHS to monitor the location of portable medical devices so adapting this method may prove cost effective and efficient in those cases. Generally, a detailed understanding of when, where and how long ICU registrars spend off-unit will assist in customising staffing in ICU and in areas where ICU expertise are required (e.g. the ED). Additionally, being aware of when and how often an ICU is without a registrar with airway expertise (as was the case for up to 10% of the night shift) is of clinical importance. Locally, this data informed staffing decisions.

## Limitations

The temporal resolution (5 min) of this study was preset by the tracking devices and could not be modified. ‘Floor hopping’, where the RTLD communicates a WAP on the floor above or below is dependent on WAP layout and occurred roughly 1% of the time. This system requires Aeroscout or similar hardware and software to be installed within the hospital and collaboration with skilled hospital Information Technology technicians. These data give ICU registrar work locations and times but not work type. We made the assumption that ICU registrars were off-unit in response to work demand alone and this may have not always been the case. Likewise, we acknowledge individual doctors will have approached the same workload in different ways. The data was anonymised so individual doctor off-unit workload patterns were not evaluated and this may have introduced bias into the results.

## Conclusions

RTLDs provide a reliable, automated method for quantifying ICU registrar off-unit work patterns. This method may be adopted for quantifying other clinical staff work patterns in suitably equipped hospital environments.

## Figures and Tables

**Fig. 2:**
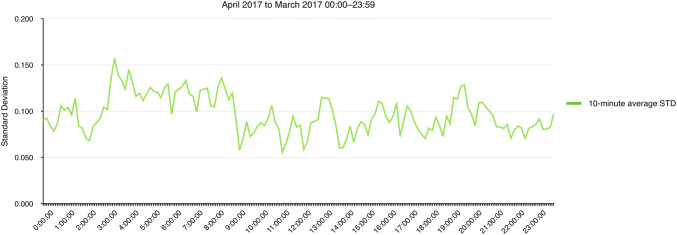
Variation (standard deviation) of the mean percentages of time spent on or off-unit. The least variability is seen around handover times (0830)

## Data Availability

The datasets generated and analysed during the current study will not be publicly available as they can be linked to the treatment of specific individuals. Aggregated data are available from the corresponding author on reasonable request.

## Appendix

See Figs. 2, 3, and 4.

## Abbreviations

ICU: Intensive Care Unit
ED: Emergency Department
EAU: Emergency Assessment Unit
RRS: Rapid Response System
RTLD: Real Time Location Device
OUHNHSFT: Oxford University Hospitals National Health Service Foundation Trust
NHS: National Health Service
